# Functional and Anatomical Changes of Acute Solar Retinopathy Investigated With Electroretinography and Optical Coherence Tomography: A Case Report

**DOI:** 10.7759/cureus.82843

**Published:** 2025-04-23

**Authors:** Korolos Sawires, Bonnie He, Jeff Locke, Jennifer Gao, R. Rishi Gupta

**Affiliations:** 1 Medicine, Faculty of Medicine, Dalhousie University, Halifax, CAN; 2 Ophthalmology and Visual Sciences, Dalhousie University, Halifax, CAN; 3 Ophthalmology, St. Martha's Regional Hospital, Antigonish, CAN

**Keywords:** electroretinography, erg, fferg, mferg, multimodal imaging, retina, solar eclipse, solar retinopathy

## Abstract

The purpose of this case is to illustrate how early electroretinography (ERG) can be used to investigate functional macular changes caused by acute solar retinopathy. This is a case report of a 44-year-old woman who presented on April 12, 2024 with acute solar retinopathy after viewing the solar eclipse on April 8, 2024 without wearing solar viewing glasses.

The patient’s best corrected visual acuity (BCVA) was 20/800 OU. Yellow macular lesions were seen on fundoscopy bilaterally. Spectral domain optical coherence tomography (SD-OCT, Spectralis; Heidelberg Engineering, Heidelberg, Germany) revealed bilateral foveal hyperreflectivity of the outer retina with attenuation of the external limiting membrane, ellipsoid zone, and retinal pigment epithelium (RPE) interdigitation line. Early ERG was performed to determine retinal function. Full field ERG (ffERG) demonstrated normal generalized retinal function OU; however, multifocal ERG (mfERG) showed bilateral macular dysfunction primarily affecting the foveal and parafoveal regions. These findings persisted at the two-month follow-up with no significant improvement in the BCVA.

Acute solar retinopathy presents with anatomical changes to the retinal layers, particularly photoreceptors. Damage and disruption to photoreceptors can result in consequent apoptosis and atrophy that can be seen on fundoscopy and OCT imaging. Measures of retinal function, such as ERG, can be used in determining baseline and progression of visual function. Additionally, this case illustrates the importance of public health measures to educate the general population about the dangers of sungazing.

## Introduction

Light can potentially cause retinal toxicity through three main mechanisms: photochemical, photothermal, and photomechanical [1]. Solar retinopathy is an acquired photochemical retinal injury that occurs secondary to direct sun gazing or eclipse viewing [2]. Free radicals generated after exposure to visible light or ultraviolet irradiation result in retinal toxicity at the level of the retinal pigment epithelium (RPE) [1,2]. Pertinent risk factors include sungazing, photosensitizing medications (e.g., thiazide diuretics, tetracycline antibiotics), psychiatric disease, and occupational exposures such as welding without appropriate equipment.

Clinically, solar retinopathy manifests as bilateral decreased visual acuity, blurred vision, central or paracentral scotomas, metamorphopsia, and dyschromatopsia [3]. While fundoscopy can reveal characteristic signs of solar retinopathy, such as central foveal yellow-white spots, optical coherence tomography (OCT) remains the most widely used modality for diagnosis [4]. Additionally, retinal function can be evaluated using electroretinography (ERG). ERG, particularly multifocal ERG (mfERG), allows us to make a more discriminative diagnosis when combined with clinical presentation and multimodal imaging [4]. Observational reports have investigated the use of steroids for treatment, but currently, there is no existing gold standard treatment for solar retinopathy [4]. As such, the prevention of solar retinopathy is the mainstay of therapy. Improvement in visual acuity has been noted to occur during the first six weeks post-exposure but can extend to the six-month mark [5-7]. Our objective is to report an early case of solar retinopathy investigated with ERG and OCT and to illustrate the role of mfERG testing in acute solar retinopathy.

## Case presentation

Clinical findings

A 44-year-old female presented to a tertiary retina clinic on April 12, 2024 with bilateral decreased vision after watching the solar eclipse on April 8, 2024, without proper viewing glasses. She reported bilateral blurred central vision and metamorphopsia. The patient did not report light sensitivity, contrast sensitivity, dyschromatopsia, or other changes in visual function beyond blurred vision and metamorphopsia. Otherwise, she had no past ocular or medical history and was not on any medications. Her best corrected visual acuity (BCVA) was 20/800 on a low vision chart in both eyes (OU). Anterior segment exam revealed normal lids, lashes, lacrimal system, sclera, and conjunctiva with a clear cornea and a deep and quiet anterior chamber bilaterally. Intraocular pressure (IOP) was measured at 16 mmHg in the right eye (OD) and 18 mmHg in the left eye (OS) via Tonopen. Fundus exam showed subtle macular lesions bilaterally, with an operculated hole temporally OD. Given the profound bilateral visual loss with relatively subtle macular changes on exam, the differential diagnosis initially included phototoxic retinopathy, hereditary maculopathies, and other diffuse retinal dysfunction. As part of the early workup, full-field ERG (ffERG) was obtained to evaluate for generalized retinal involvement and to guide diagnosis.

The patient was seen for follow-up approximately two months later on June 3, 2024. The patient’s BCVA was 20/800 OD and 20/600 OS. Her symptoms remained the same, with no reported improvement in blurred vision or metamorphopsia. Anterior segment examination was unremarkable, and IOP measured 13 mmHg OD and 17 mmHg OS via Tonopen. Fundus examination revealed larger and more distinct macular lesions bilaterally.

Imaging and functional testing

**Figure 1:**
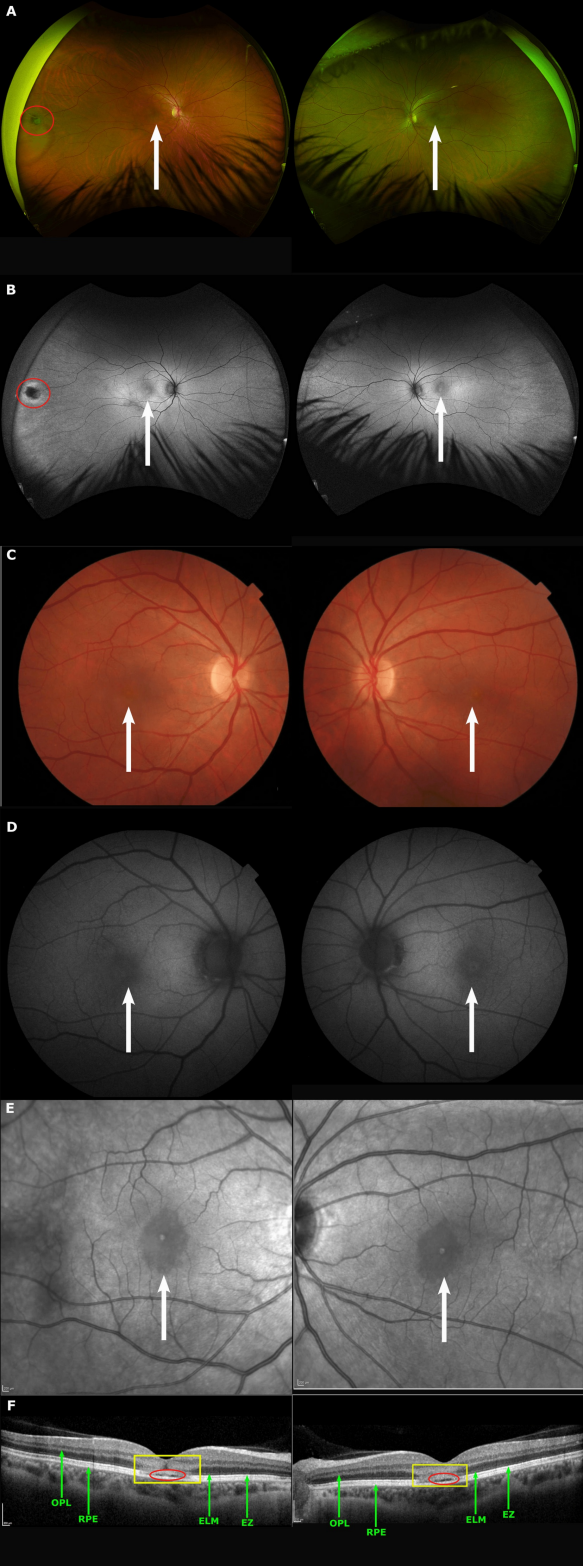
Multimodal imaging from initial presentation (April 12, 2024) (A) Ultrawide field pseudocolor fundus. (B) Ultrawide field green fundus autofluorescence demonstrating subtle foveal lesions OU (white arrows) and an operculated hole temporally OD (red circle). (C) Color fundus. (D) Fundus autofluorescence. (E) Near-infrared imaging reveals foveal lesions more clearly (white arrows). (F) Spectral domain optical coherence tomography (SD-OCT) illustrating profound bilateral foveal hyperreflectivity (yellow rectangle), extending from the outer plexiform layer (OPL; green arrow) to the retinal pigment epithelium (RPE; green arrow) with attenuation of the external limiting membrane (ELM; green arrow), ellipsoid zone (EZ; green arrow), and RPE interdigitation line resulting in a hypo-reflective space (red circle).

Multimodal imaging was performed at initial presentation (April 12, 2024). On dilated examination, fundus photographs (Figures 1A-1C) (Optos, Dunfermline, Scotland) revealed subtle yellowish alterations in the fovea [8]. There was an operculated hole surrounded by subtle RPE changes temporally OD (Figure 1A). Fundus autofluorescence imaging (Figure 1D) showed subtle foveal hyper-autofluorescence. The near-infrared imaging (Figure 1E), however, showed a larger area of involvement, with symmetric foveal hypo-reflective lesions of approximately one disc diameter in size. Spectral domain optical coherence tomography (SD-OCT, Spectralis; Heidelberg Engineering, Heidelberg, Germany) of the macula demonstrated a preserved foveal contour with bilateral foveal hyperreflectivity, extending from the outer plexiform layer (OPL) to the retinal pigment epithelium (RPE), as delineated by the yellow lines (Figure 1F) [9]. There was attenuation of the external limiting membrane (ELM), ellipsoid zone (EZ), and RPE interdigitation line, yielding a subtle hypo-reflective space above the RPE line (red circles; Figure 1F).

**Figure 2:**
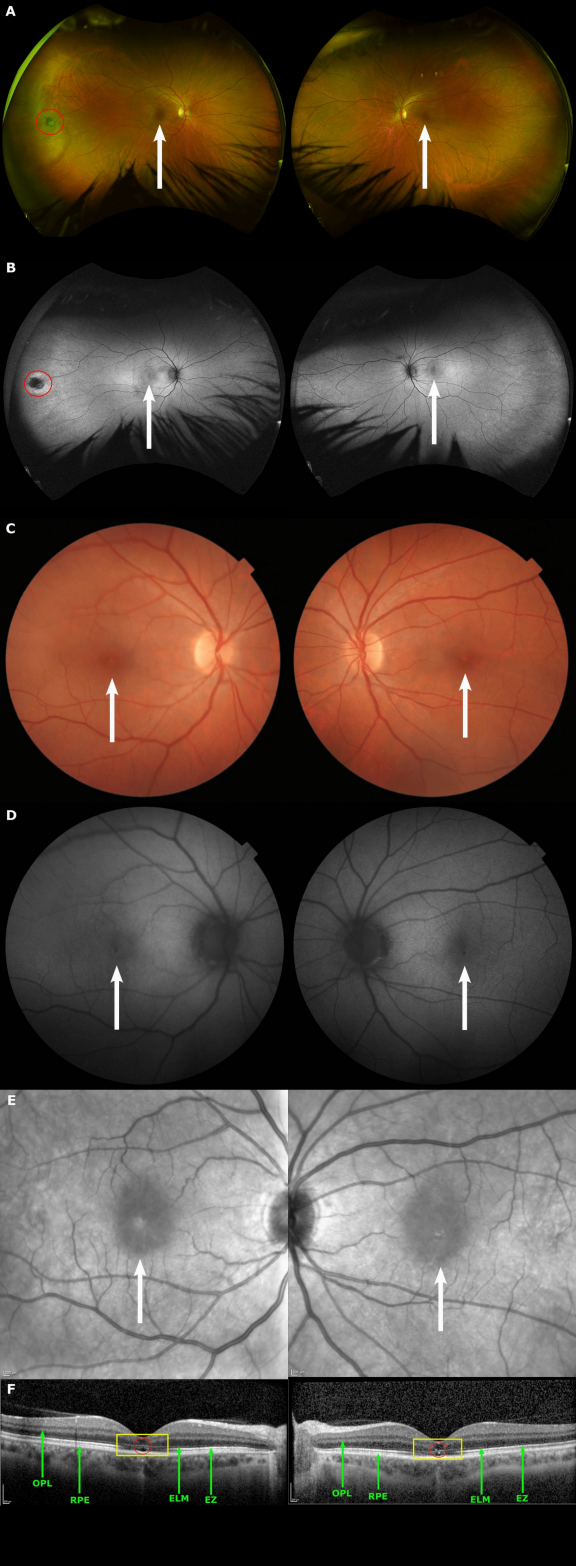
Multimodal imaging from follow-up two months post presentation (June 3, 2024) (A) Ultrawide field pseudocolor fundus. (B) Ultrawide field green fundus autofluorescence demonstrating subtle foveal lesions OU (white arrows) and an operculated hole temporally OD (red circle). (C) Color fundus. (D) Fundus autofluorescence. (E) Near-infrared imaging reveals foveal lesions more clearly (white arrows). (F) Spectral domain optical coherence tomography (SD-OCT) illustrating profound bilateral foveal hyperreflectivity (yellow rectangle), extending from the outer plexiform layer (OPL; green arrow) to the retinal pigment epithelium (RPE; green arrow) with attenuation of the external limiting membrane (ELM; green arrow), ellipsoid zone (EZ; green arrow), and RPE interdigitation line resulting in a hypo-reflective space (red circle).

Repeat fundus photography, SD-OCT, and mfERG were completed at follow-up on June 3, 2024, approximately two months after the patient's initial presentation. Color fundus, fundus autofluorescence, and near-infrared photographs show larger and more clearly demarcated foveal lesions compared to photos from initial presentation (Figures 2A-2E). SD-OCT imaging revealed significant resolution of the outer retinal hyperreflectivity (Figure 2F). There was moderate reconstitution of the ELM and EZ, however, the foveal RPE interdigitation line remained quite attenuated, with resultant increase in the size of the hypo-reflective space above the RPE line (red circles; Figure 2F). The central outer nuclear layer (ONL) appeared thinned secondary to atrophy (Figure 2F).

**Figure 3:**
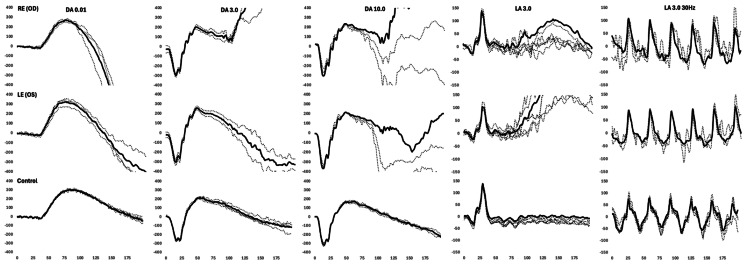
International Society for Clinical Electrophysiology of Vision (ISCEV) full field electroretinogram (ERG) of the right eye (OD), left eye (OS), and normative representative traces (control). Recordings obtained using Dawson, Trick, and Litzkow (DTL) electrodes and ColorDome Ganzfeld (Diagnosys LLC, Lowell, USA). All major peaks (a-waves, b-waves, 30Hz flicker peaks) were within the limits of normality for age-matched normative data for both amplitude (µV) and implicit timing (ms).

**Figure 4:**
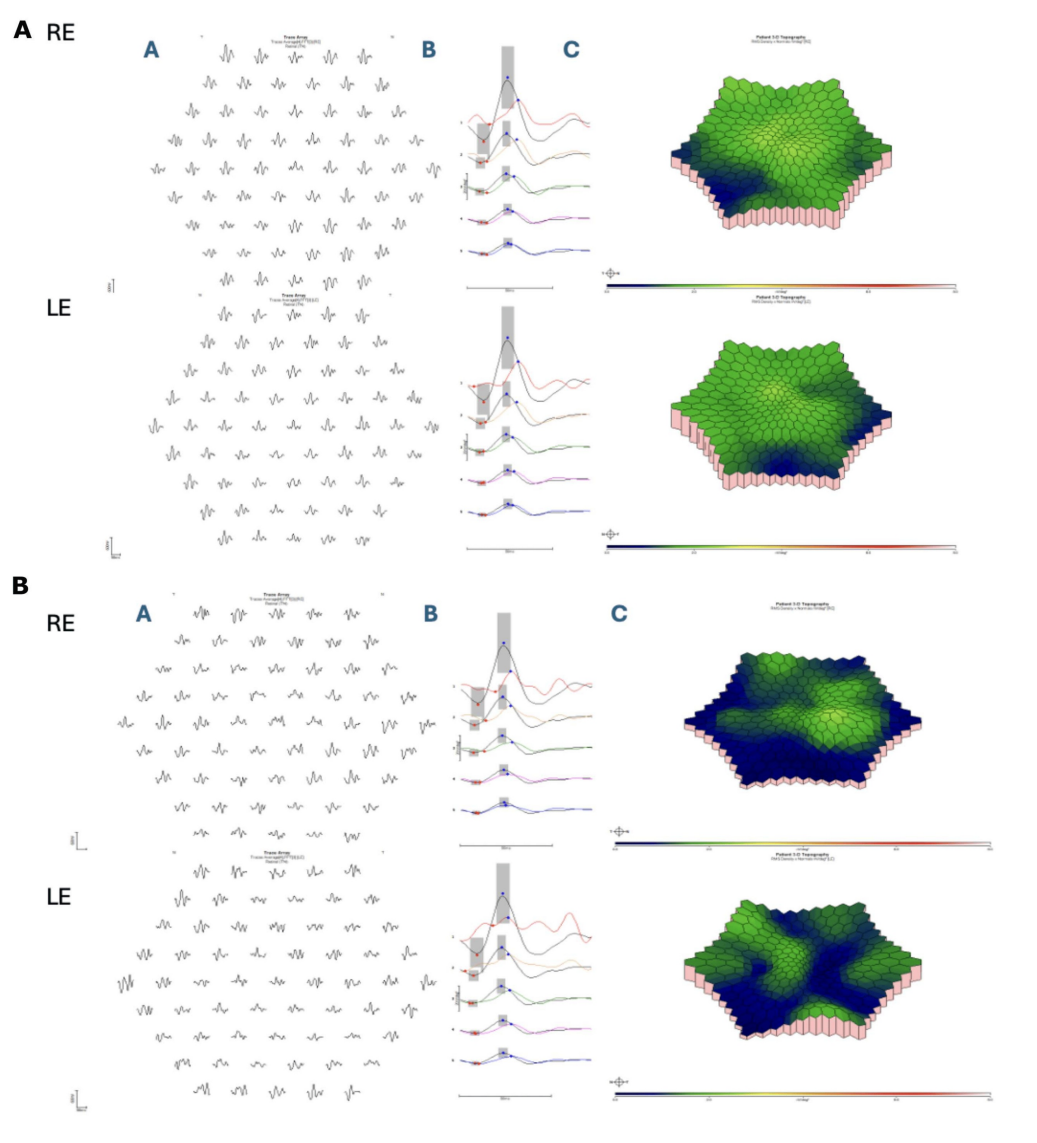
International Society for Clinical Electrophysiology of Vision (ISCEV) 61 hexagon multifocal electroretinogram (mfERG) Initial visit (A, top half): (A) Raw traces obtained from different eccentricities with solid (black) lines representing patient waveforms (OD top, OS bottom). (B) Ring-averages from mfERG traces of concentric hexagons averaged across five different eccentricity ranges, with solid black lines representing normative data, and colored lines representing patient data. Normative amplitude and implicit timing within two standard deviations (gray box) for P1(nV/d2). (C) 3D-response density plot (retina view-OD top, OS bottom). Recordings were obtained using Dawson, Trick, and Litzkow (DTL) electrodes and an LCD monitor (Diagnosys LLC, Lowell, USA). The central foveal P1(nV/d2) responses were mild-moderately reduced with significantly delayed implicit timing in either eye. Parafoveal rings showed normal age-matched amplitudes with significantly delayed implicit timing in either eye. Perifoveal rings showed normal age-matched amplitudes with borderline implicit timing. Follow-up visit (B, bottom half): (A) Raw traces obtained from different eccentricities with solid (black) lines representing patient waveforms (OD top, OS bottom). (B) Ring-averages from mfERG traces of concentric hexagons averaged across five different eccentricity ranges, with solid black lines representing normative data, and colored lines representing patient data. Normative amplitude and implicit timing within two standard deviations (gray box) for P1(nV/d2). (C) 3D-response density plot (retina view: OD top, OS bottom). The central foveal P1(nV/d2) responses remain moderately reduced (OS more reliable) with borderline implicit timing in either eye. Parafoveal rings were borderline in amplitude with delayed implicit timing. Perifoveal rings continue to show normal age-matched amplitudes with delayed implicit timing in the RE only. Compared to the baseline mfERG, the amplitudes remain reduced within the foveal/parafoveal regions, with some improvements in implicit timing.

As part of the patient’s ophthalmic workup, a full field electroretinogram (ffERG) was completed to rule out general retinal dysfunction (Figure 3), and a multifocal electroretinogram (mfERG) to quantify macular function (Figure 4A), by the International Society for Clinical Electrophysiology of Vision (ISCEV) standards [10,11]. Electrodiagnostic testing was recorded binocularly using Dawson, Trick, and Litzkow (DTL: Diagnosys LLC, Lowell, MA, USA), ipsilateral outer canthus references electrodes (Genuine Grass Gold cup electrodes: Natus Medical Inc., Middleton, WI, USA), and a common ground electrode (Genuine Grass) placed on the forehead. The pupils were fully dilated with 1% tropicamide and electrodiagnostic recordings obtained using a ColorDome (Diagnosys LLC) Ganzfeld (ERG) and 27 27-inch Liquid Crystal Display (LCD) (Estecom, Gyeonggi, Korea) screen (mfERG), Espion Analysis Software (Software version V6.66.1: Diagnosys LLC) [12-14]. The patient was dark adapted (DA) for 20 minutes and presented with flash intensities of DA 0.01, 3.0 and 10.0 cd·s/m^2^ (candela second per square meter), respectively, and then light adapted (LA) for 10 minutes, followed by presentation of a LA 3.0 cd·s/m^2^ flash and LA 3.0 30Hz flicker stimulus. Following LA ERG testing, a 61 hexagonal array mfERG (14 m-sequence bits) subtending 50 degrees of central retina was completed in standard room illumination, with ongoing patient fixation monitored via desktop fixation camera (Diagnosys LLC). The ERG in either eye was within normal limits (lab-based - age-matched controls) for all major peaks, in regard to amplitude (µV), implicit timing (ms), and waveform morphology, to all tested DA and LA stimuli (Figure 4A). Conversely, the mfERG central foveal P1(nV/d2) responses were mild-moderately reduced with significantly delayed implicit timing in either eye. Parafoveal rings showed normal age-matched amplitudes with significantly delayed implicit timing in either eye. Perifoveal rings showed normal age-matched amplitudes with borderline implicit timing (Figure 4A, Table 1).

**Table 1:** International Society for Clinical Electrophysiology of Vision (ISCEV) 61 Hexagon mfERG response density and implicit timing ISCEV Multifocal electroretinogram 61 hexagon array (14-Sequence Bits) Group Averages with response density (nV/d2: nanovolt per degree squared) and implicit timing (ms) for major peak (P1) in the right eye (OD) and left eye (OS) for initial visit (visit 1) and 8 week follow up (visit 2). Values provided for each ring (area: degree squared) of eccentricity away from fixation. Percent difference of follow-up P1 nV/d2 amplitudes in OD (right eye) and OS (left eye) compared to baseline (April 2024) mfERG, with negative values indicating a decrease from baseline.

Response density												
			Visit 1				Visit 2				% Change	
Index	Degrees	Area d^2^	OD P1 (ms)	OD P1 (nV/d^2^)	OS P1 (ms)	OS P1 (nV/d^2^)	OD P1 (ms)	OD P1 (nV/d^2^)	OS P1 (ms)	OS P1 (nV/d^2^)	OD P1	OS P1
1	0.0	15.0	37.1	18.8	37.1	19.0	33.8	6.2	34.6	16.2	-67	-15
2	4.5	20.9	36.3	17.1	36.3	15.4	33.8	13.2	34.6	11.7	-23	-24
3	9.6	29.4	34.6	12.8	33.8	10.7	34.6	10.1	35.4	7.2	-21	-33
4	15.9	38.3	33.8	8.9	34.6	8.5	35.4	7.9	32.9	6.5	-11	-24
5	23.0	46.1	32.9	8.2	33.8	7.9	35.4	5.8	32.1	6.6	-29	-16
Reliability Index			100%		100%		100%		100%		P1 nV/d^2^ % change	
Total blinks recorded			8		8		35		35			

**Figure 5:**
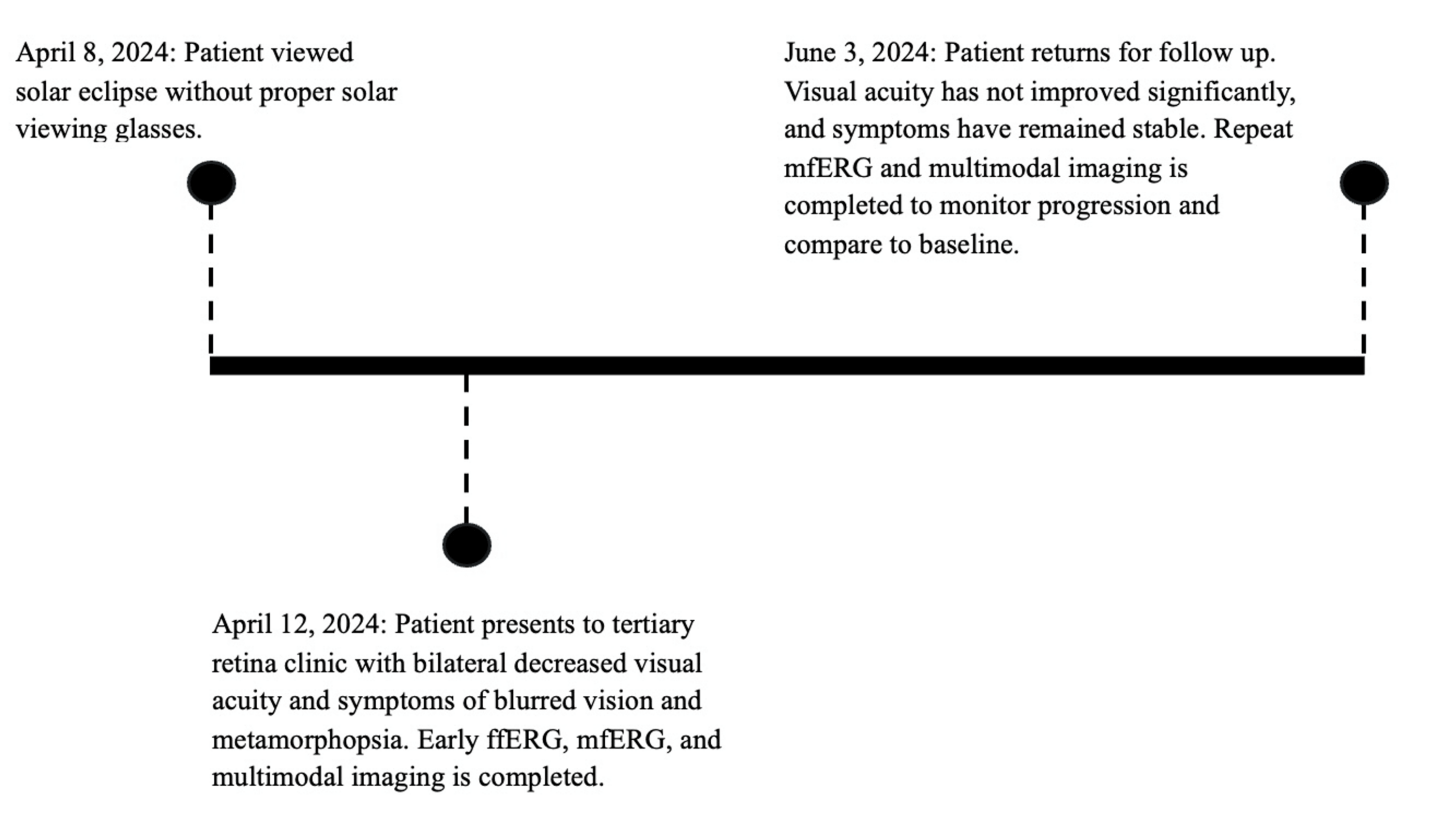
Visual timeline of patient care

Results from the mfERG showed central foveal P1(nV/d2) responses remained moderately reduced (OS more reliable) with borderline implicit timing in either eye. Parafoveal rings were borderline in amplitude with delayed implicit timing. Perifoveal rings continue to show normal age-matched amplitudes with delayed implicit timing in the right eye (OD) only. Compared to the baseline mfERG, the amplitudes remained reduced within the fovea/parafovea with some improvements in implicit timing (Figure 4B, Table 1). A visual timeline of the patient's care is presented in Figure 5. 

## Discussion

Anatomical changes

Anatomical changes in the retina can be seen early after sun exposure. Classic yellow-white spots in the fovea can become more distinct over time on fundoscopy. Typical OCT findings include foveal hyperreflectivity in the outer retinal layers and a hypo-reflective foveal defect at the level of the EZ extending to the RPE [15]. It is thought that the hyperreflective regions are related to photoreceptor damage and disruption, while the hypo-reflective spaces represent areas of photoreceptor apoptosis and atrophy [16]. This coincides with follow-up SD-OCT imaging, whereby hypo-reflective spaces became larger and foveal hyperreflectivity decreased.

Functional changes

Although BCVA and visual field testing are the more common modes of assessing ocular function, ERG represents another modality to objectively evaluate retinal function [4]. Previous case reports have examined mfERG-guided functional changes associated with chronic solar retinopathy [2,3]. Stangos et al. reported two cases of solar retinopathy using mfERG years after their diagnosis. One patient had markedly reduced mfERG response amplitudes in the central 20 degrees OU, while the other patient had moderately reduced amplitudes in the central 10 degrees OU [3]. Similarly, Schatz et al. investigated two patients with a 241 Hexagon mfERG two months after their diagnosis. The first patient had diminished mfERG responses OD compared to OS, while the other had central retinal dysfunction OU. At follow-up, mfERG showed improved function for both patients [2]. It is important to note that to obtain reliable readings with 241 Hexagon mfERG, subjects must be able to fixate and concentrate throughout the examination, which is often a problem in patients with macular dysfunction. As such, it is not commonly used in the clinic. Banda et al. reported a case of acute solar retinopathy in a patient who presented four days after eclipse viewing. The patient was investigated using fluorescein angiography (FA), OCT, and mfERG. mfERG was conducted one week and one month after the initial presentation. The BCVA was noted to be 20/200 OU; the patient was initiated on oral methylprednisolone and topical difluprednate BID. Fortunately, their vision improved to 20/70 OD and 20/100 OS. Details of mfERG were not given, but revealed a general improvement of central P1 and N1 responses at the one-month follow-up. No ffERG was completed to rule out general retinal dysfunction [17].

While mfERG has been used in the setting of chronic solar retinopathy, it is not well documented in the acute phase of solar retinopathy. In our patient, early ERG and mfERG, four days after sun exposure, illustrated normal generalized retinal function in both eyes with bilateral macular dysfunction primarily affecting the fovea and parafovea. Two months later, during follow-up, mfERG continued to demonstrate attenuated responses with improvements in the implicit timing. These results were not attributed to poor fixation. This case highlights the utility of early mfERG in diagnosing acute solar retinopathy, especially in patients presenting with severe visual loss. By confirming localized macular dysfunction with preserved peripheral retinal function, mfERG can support clinical diagnosis and guide patient counseling. Early electrodiagnostic testing may be underutilized in acute settings and warrants greater consideration in similar presentations.

Limitations

Firstly, as a single case report, a small sample size (n=1) limits the generalizability of our findings. However, the correlation between structural imaging and functional testing provides meaningful insight into the diagnostic utility of mfERG in acute solar retinopathy. Secondly, while mfERG was successfully obtained at both time points with high reliability indices, some technical considerations should be noted. The second session had a higher blink rate, introducing mild waveform noise, particularly in the right eye. Although testing parameters were standardized (consistent pupil dilation, electrode impedance, and the use of the same experienced examiner), minor positional changes of the DTL electrode and the inherent inter-visit variability of mfERG may have influenced the amplitude differences observed [18]. As such, the apparent reduction in central P1 amplitude in the right eye should be interpreted with caution and in conjunction with clinical and imaging findings. Lastly, no interventional therapy was initiated, so functional improvements could not be evaluated under treatment conditions.

## Conclusions

In summary, we present multimodal imaging of a case of acute solar retinopathy with early mfERG assessment following exposure. Acute solar retinopathy presents with anatomical changes to the retinal layers, particularly photoreceptors. Damage and disruption to photoreceptors can result in consequent apoptosis and atrophy that can be seen on fundoscopy and OCT imaging. Measures of retinal function, such as ERG, may be underutilized in acute settings but can be useful in determining baseline and progression of visual function. As such, ERG warrants greater consideration, particularly in patients with acutely profound visual acuity changes and subtle anatomical changes. Our patient did not undergo any treatment and, unfortunately, did not have visual acuity gains at the two-month follow-up. This case highlights the importance of public health measures to educate the general population about the dangers of sungazing.
